# Pathogenic Characterization of *Clostridium perfringens* Strains Isolated From Patients With Massive Intravascular Hemolysis

**DOI:** 10.3389/fmicb.2021.713509

**Published:** 2021-07-27

**Authors:** Ai Suzaki, Kaori Ohtani, Shihoko Komine-Aizawa, Asami Matsumoto, Shigeru Kamiya, Satoshi Hayakawa

**Affiliations:** ^1^Division of Microbiology, Department of Pathology and Microbiology, Nihon University School of Medicine, Tokyo, Japan; ^2^Division of General Medicine, Department of Medicine, Nihon University School of Medicine, Tokyo, Japan; ^3^Division of Host Defense Mechanism, Department of Bacteriology and Bacterial Infection, Tokai University School of Medicine, Isehara, Japan; ^4^R&D Division, Miyarisan Pharmaceutical Co., LTD., Saitama, Japan; ^5^Department of Infectious Diseases, Kyorin University School of Medicine, Tokyo, Japan

**Keywords:** *Clostridium perfringens*, sepsis, hemolysis, perfringolysin O, phospholipase C, cytokine

## Abstract

Sepsis caused by *Clostridium perfringens* infection is rare but often fatal. The most serious complication leading to poor prognosis is massive intravascular hemolysis (MIH). However, the molecular mechanism underlying this fulminant form of hemolysis is unclear. In the present study, we employed 11 clinical strains isolated from patients with *C. perfringens* septicemia and subdivided these isolates into groups H and NH: septicemia with (*n* = 5) or without (*n* = 6) MIH, respectively. To elucidate the major pathogenic factors of MIH, biological features were compared between these groups. The isolates of two groups did not differ in growth rate, virulence-related gene expression, or phospholipase C (CPA) production. Erythrocyte hemolysis was predominantly observed in culture supernatants of the strains in group H, and the human erythrocyte hemolysis rate was significantly correlated with perfringolysin O (PFO) production. Correlations were also found among PFO production, human peripheral blood mononuclear cell (PBMC) cytotoxicity, and production of interleukin-6 (IL-6) and interleukin-8 (IL-8) by human PBMCs. Analysis of proinflammatory cytokines showed that PFO induced tumor necrosis factor-α (TNF-α), IL-5, IL-6, and IL-8 production more strongly than did CPA. PFO exerted potent cytotoxic and proinflammatory cytokine induction effects on human blood cells. PFO may be a major virulence factor of sepsis with MIH, and potent proinflammatory cytokine production induced by PFO may influence the rapid progression of this fatal disease caused by *C. perfringens*.

## Introduction

*Clostridium perfringens* is an anaerobic spore-forming Gram-positive bacillus widely distributed in the intestinal tracts of humans and animals and in soil, and it is highly pathogenic to humans and animals (Uzal et al., 2014). *Clostridium perfringens* is classified into seven types, A–G, based on production of the six major toxins: *α*, *β*, *ε*, and *ι* toxins, enterotoxin (CPE), and necrotic enteritis toxin (NetB2). *Clostridium perfringens*, isolated mainly from humans, is classified into two types: type A, producing only *α*-toxin (phospholipase C, CPA), and type F, producing CPA and CPE. *Clostridium perfringens* secretes many other toxins and enzymes, more than 20 of which are estimated to be pathogenicity-related (Kiu and Hall, 2018).

In humans, *C. perfringens* resides in the gastrointestinal tract and genital organs and is well-known as a causative agent of mass food poisoning (Kiu and Hall, 2018), gas gangrene (Awad et al., 2001; Uzal et al., 2014), liver abscess, and sepsis (Hübl et al., 1993; van Bunderen et al., 2010; Chinen, 2020). Food poisoning develops when CPE is produced by this microorganism (Kiu and Hall, 2018). Myonecrosis in gas gangrene is caused specifically by CPA, which has phospholipase and sphingomyelinase activities, and CPA is widely accepted to be the main virulence factor. In addition, complementation with the pore-forming θ-toxin (perfringolysin O, PFO) aggravates muscle necrosis; thus, the pathogenesis of gas gangrene is currently believed to be caused by the synergistic effect of both toxins (Awad et al., 2001; Uzal et al., 2014). The incidence rate of *C. perfringens* sepsis is lower than that of food poisoning or gas gangrene, but its prognosis is very poor, especially when massive intravascular hemolysis (MIH) occurs (Chinen, 2020). *Clostridium perfringens* sepsis with MIH remains an extremely serious disease, with a fatality rate of 74–80% (van Bunderen et al., 2010; Simon et al., 2014). Because of the fulminant course of sepsis with MIH, the average hospital stay is only approximately 8 h, and most patients are in shock or dead when the diagnosis is confirmed by blood culture (van Bunderen et al., 2010).

We retrospectively compared the clinical presentation of patients with *C. perfringens* sepsis in groups with and without MIH. In our previous study, patients in the MIH group were significantly younger than those in the non-hemolysis group and more often presented with severe pain, disorientation, shock, hematuria, liver dysfunction, and metabolic acidosis on arrival at the hospital (unpublished data). All patients in the MIH group had developed systemic inflammatory response syndrome (SIRS), and all died within 26 h because of rapidly progressing lung injury and acute respiratory distress syndrome (ARDS). Older patients with sepsis exhibit a more severe clinical course and higher mortality than younger patients because of impaired cellular and humoral immunity (Martín et al., 2017). The high prevalence of SIRS/ARDS and the younger age, which increased the likelihood of an immune response, in the MIH group, strongly suggested that a strong host response is activated in patients with sepsis with MIH early in the infection course, resulting in an excessive immune response including cytokine storm (unpublished data).

The major virulence factor of sepsis with MIH is thought to be CPA, a pathogenic toxin of *C. perfringens* type A (Hübl et al., 1993). However, since all *C. perfringens* strains produce CPA, it is difficult to explain this fatal and rapidly progressive condition by the presence or absence of CPA alone. No study has conclusively identified the main virulence factor of sepsis with MIH. *Clostridium perfringens* sepsis with MIH remains a fatal disease for which major virulence factors are unknown because of its infrequent occurrence and the difficulty of collecting cases and strains due to the fulminant disease course and the common death of patients before diagnosis.

This study is the first to compare the hemolysis of human erythrocytes and the effects on human peripheral blood mononuclear cells (PBMCs) between clinical *C. perfringens* strains isolated from patients with MIH (group H) or without MIH (group NH) to elucidate the mechanism of lethal MIH in *C. perfringens* sepsis.

## Materials and Methods

### Bacterial Strains

The bacterial strains used were 11 clinical blood-derived isolates of *C. perfringens* stored at Nihon University Hospital. Of the 11 strains, five were from group H, whose sera appeared exceptionally bright red with marked hemoglobinemia on arrival at the hospital (Chinen, 2020), and six were from group NH. The clinical backgrounds of the isolates used in this study are provided in Table 1. *Clostridium perfringens* standard strain 13, isolated from a patient with gas gangrene (Shimizu et al., 2002), was used as the reference strain. The study was approved by the ethical committee of Nihon University Hospital (Number 20160401).

**Table 1:** Clinical profiles of 11 blood-derived *Clostridium perfringens* isolates used in this study.

Isolate	Massive intravascular hemolysis^*^	Clinical conditions	Source of bacteremia	Onset place of infection	Gas formation on imaging examination	Cause of death	Length of hospital stay from bacteremia onset to death
H1	+	Diarrhea from 3 days before shock and multiple organ failure	Intra-abdominal	Community-acquired	−	Sepsis	1 h
H2	+	Back pain from 2 days before pulmonary hemorrhage with cholangiocarcinoma	Hepatobiliary tract	Community-acquired	−	Sepsis	2.5 h
H3	+	Under chemotherapy for carcinosarcoma of ovary	Genital tract	Community-acquired	+	Sepsis	6 h
H4	+	Traffic bruise 10 days before chest pain	Trauma	Community-acquired	+	Sepsis	10 h
H5	+	Right hypochondralgia	unknown	Community-acquired	+	Sepsis	26 h
NH1	−	Stool extraction the day before	Intra-abdominal	Nosocomial	−	Chronic heart failure	20 days
NH2	−	Acute cholecystitis	Hepatobiliary tract	Nosocomial	−		
NH3	−	Ascites with liver failure	Intra-abdominal	Community-acquired	−		
NH4	−	Pneumonia using glucocorticoid for asthma	Respiratory tract	Nosocomial	−		
NH5	−	Carcinomatous pleurisy of lung cancer	Respiratory tract	Community-acquired	−		
NH6	−	Necrotizing cholecystitis and diffuse peritonitis	Hepatobiliary tract	Community-acquired	+		

* Whose serum appeared exceptionally bright red with marked hemoglobinemia.

### Culture Conditions and Collection of Culture Supernatant

Frozen clinical isolates were first recovered on sheep blood agar under anaerobic conditions overnight at 37°C and were then cultured in Gifu anaerobic medium (GAM) broth (Nissui, Tokyo, Japan) for 12 h to stationary phase (Ohtani et al., 2009). These strains were washed twice, inoculated at a 1% concentration in fresh GAM medium, and cultured anaerobically at 37°C. An aliquot of each culture was removed every 1 h and bacterial growth was measured by change of aliquots absorbance at 600 nm using an absorbance microplate reader iMark microplate reader (Bio-Rad, United Kingdom). Growth curves were plotted for each isolate. *Clostridium perfringens* supernatants were collected at a concentration of 6 × 10^6^ cfu/ml in early logarithmic growth phase, centrifuged at 19,370 *g* for 5 min, and then the supernatants were sterile-filtered (pore size 0.22 μm; Merck Millipore, Burlington, MA, United States).

### DNA Manipulation

All strains were cultured in GAM broth and used to isolate chromosomal DNA. The *C. perfringens* chromosomal DNA library was constructed as described previously (Ohtani et al., 2010). PCR was performed using primers and EX Taq (Takara Bio, Shiga, Japan) with total DNA from each strain (Supplementary Table S1).

### Isolation of Human Erythrocytes and PBMCs

Heparinized human peripheral blood samples were collected from five healthy adult volunteers with informed consent on the day of the experiment. Human PBMCs were prepared within 3 h after blood collection with Lymphoprep™ (Axis-Shield, Norway). The study was approved by the institutional review board of Nihon University School of Medicine (Number 30-13-1).

### Hemolysis Assays

Hemolysis assays were performed according to the method of Larsen et al. (2011). Erythrocytes were washed three times with phosphate buffered saline (PBS) and mixed to yield suspensions containing 40% (*v*/*v*) erythrocytes, corresponding to the approximate human hematocrit value, and 50% (*v*/*v*) clinical isolate culture supernatants. The suspensions were incubated at 37°C for 60 min, and the absorbance of the supernatants was detected at 540 nm as a measure of hemoglobin release. GAM broth was used as the control and the level of hemolysis was calculated by the following equation: *Hemolysis* = (*Abs*_sample_ − *Abs*_control_)/(*Abs*_max lysis_ − *Abs*_control_) × 100.

The hemolysis assay was performed in three independent experiments with technical triplicates.

### Measurement of CPA in Culture Supernatants of Clinical Isolates by Quantitative Enzyme-Linked Immunosorbent Assay

For the detection of CPA in the supernatants of clinical isolates, a Monoscreen AgELISA *C. perfringens* α-Toxin Kit (Bio-X Diagnostics S.A., Rochefort, Belgium) was used according to the instructions of the manufacturer. The amount of CPA was calculated using quantitative enzyme-linked immunosorbent assay (ELISA) with a standard (CPA from *C. perfringens* type I; Sigma-Aldrich, St. Louis, MO, United States), as described previously (Zhang et al., 2006). The CPA ELISA was performed in three independent experiments with technical duplicates.

### Detection of PFO in Culture Supernatants of Clinical Isolates by Western Blot Analysis

The culture supernatant of each clinical isolate was mixed with sodium dodecyl sulfate (SDS) loading buffer and boiled for 10 min (Li et al., 2011). Those mixtures were electrophoresed on a pre-cast gel (NuPAGE 4–12% Bis-Tris Gel; Invitrogen, Carlsbad, CA, United States) and separated by electrophoresis. Western blot analysis was performed using a rabbit anti-PFO antibody (Abcam, Cambridge, United Kingdom) and visualized with a Luminescent Image Analyzer, Image Reader LAS-4000 mini (Fujifilm K. K., Tokyo, Japan). Four separate experiments were performed for analysis. The amount of PFO produced in each individual preparation was normalized to the amount produced by strain 13 as the control.

### Cytotoxicity Assay in PBMCs

Human PBMCs were collected from five healthy donors on the day of the experiment and suspended in RPMI 1640 medium (Gibco-BRL) supplemented with 10% fetal bovine serum, 1.0 mg/ml streptomycin, and 50 IU/ml penicillin to a final concentration of 1 × 10^6^ viable cells/ml. Then, 100 μl of the PBMC suspension and supernatants of clinical isolates or recombinant toxins were added to 96-well plate and cultured with 5% CO_2_ at 37°C for 4 h. The number of viable cells was determined using a cell counting kit-8 (CCK-8; Dojindo, Kumamoto, Japan; Li et al., 2020). GAM broth or PBS was used as a control, and the percentage of the cytotoxicity was calculated by the following equation: *Cytotoxicity* = (*Abs*_control_ − *Abs*_sample_)/(*Abs*_control_) × 100.

The cytotoxicity assay was performed in three independent experiments with technical triplicates.

### Stimulation of PBMCs

Peripheral blood mononuclear cells were stimulated by adding bacterial supernatant, recombinant toxins [recombinant PFO (rPFO), Cusabio Technology; recombinant CPA (rCPA): CPA from *C. perfringens* type I, Sigma-Aldrich, St. Louis, MO, United States], and GAM or PBS as a negative control. The plates were incubated at 37°C in a 5% CO_2_ atmosphere for 4 h, and the supernatants were collected after centrifugation (8,500 × *g*, 5 min) and stored at −20°C (Tuovinen et al., 2013).

### Measurement of Interleukin-6 and Interleukin-8 Production by PBMCs

The culture supernatant of each clinical isolate was added separately to PBMCs to a final concentration of 1%. After 4 h of incubation, the concentrations of interleukin-6 (IL-6) and interleukin-6 (IL-8) in the PBMC culture medium were determined by ELISA (Proteintech) and were then normalized to the number of PBMCs and compared between groups H and NH. The cytokine sandwich ELISA protocol was followed according to the instructions of the manufacturer (Tuovinen et al., 2013). The IL-6 and IL-8 ELISAs were performed in three independent experiments with technical duplicates.

### Measurement of Inflammatory Cytokine Production by PBMCs

Peripheral blood mononuclear cells were stimulated with 0.016 U/ml rCPA and 63 ng/ml rPFO. As a control, PBMCs were treated with PBS for 4 h. Cytokine concentrations were measured using a Bio-Plex Pro Human Th17 Cytokine Assay Kit (Bio-Rad Laboratories, Hercules, CA, United States) according to the instructions of the manufacturer (Komine-Aizawa et al., 2015) and normalized to the number of PBMCs.

### Statistical Analysis

The data were analyzed using the Mann–Whitney test, Pearson correlation analysis, and the Tukey–Kramer test using Statcel 3 software (The Publisher OMS Ltd., Tokorozawa, Saitama, Japan). A probability value of < 0.05 was considered to indicate a significant difference.

## Results

### Gene Variations in Possible Virulence Factors in Blood-Derived *Clostridium perfringens* Clinical Isolates

To compare the gene variations in possible virulence factors between clinical isolates in groups H and NH, known virulence-related genes were examined based on the gene repertoire of *C. perfringens* strain 13 generated by whole-genome sequencing (Supplementary Table S2). The gene variations in possible virulence factors did not differ between the five strains in group H and the six strains in group NH.

### Proliferation of Blood-Derived Clinical Isolates

**Figure 1:**
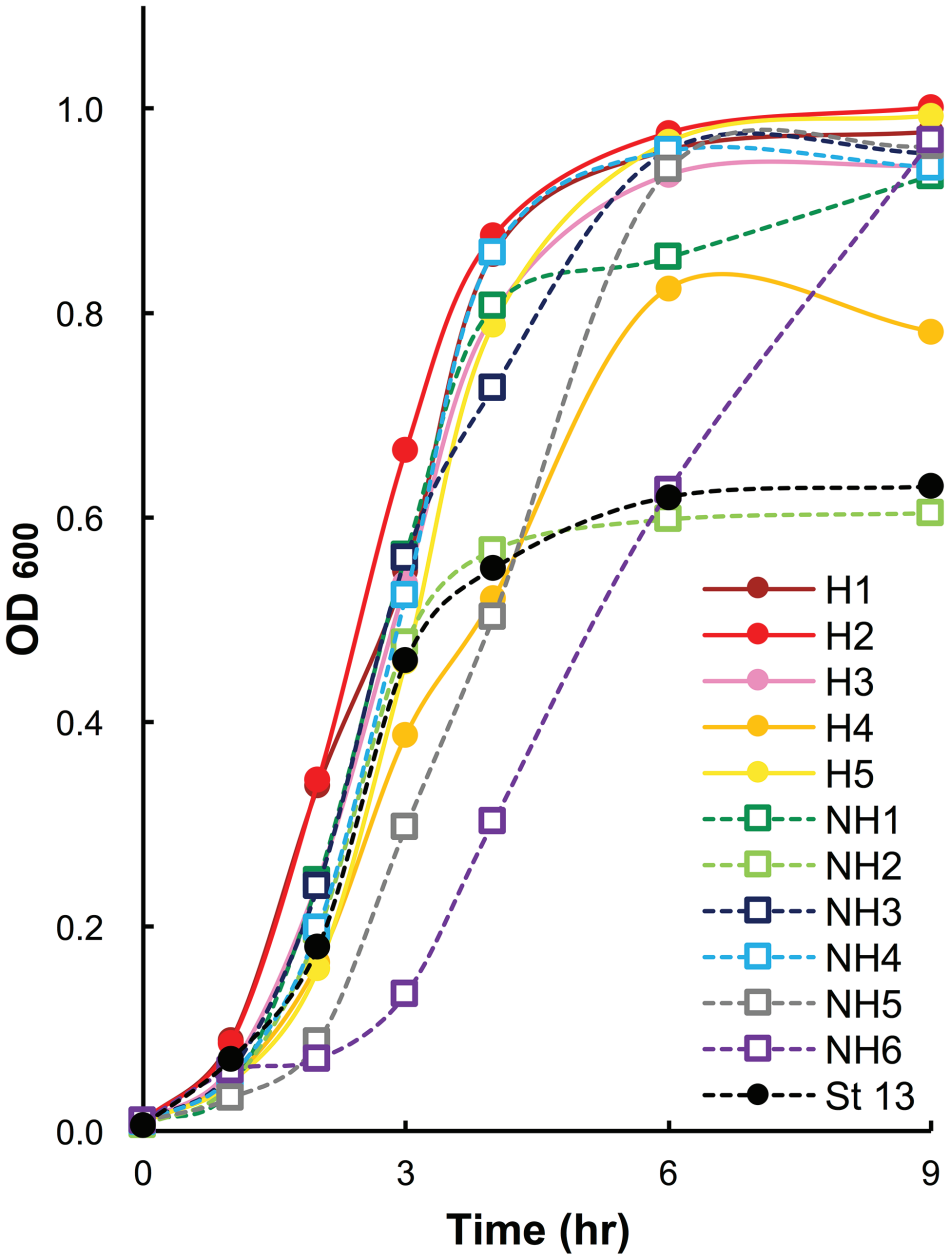
Growth curves of blood-derived clinical isolates. Clinical isolates in stationary phase were inoculated at a 1% concentration, and bacterial growth was assessed by measuring the change in the absorbance of the aliquots at 600 nm. OD_600_: optical density at 600 nm. In the graph, strains in group H are shown with solid circles and solid lines, strains in group NH with open squares and dashed lines, and strain 13 with black markers and a dashed line. Group H, massive intravascular hemolysis group; Group NH, nonhemolysis group.

The clinical isolates in group H tended to grow faster than those in group NH, but no significant difference was observed between the two groups (Figure 1). Since there were two particularly slow-growing isolates (NH5 and NH6) in group NH, nine isolates, excluding these two isolates, were used for further investigations.

### Hemolytic Effect of Culture Supernatants of Clinical Isolates on Human Erythrocytes

**Figure 2:**
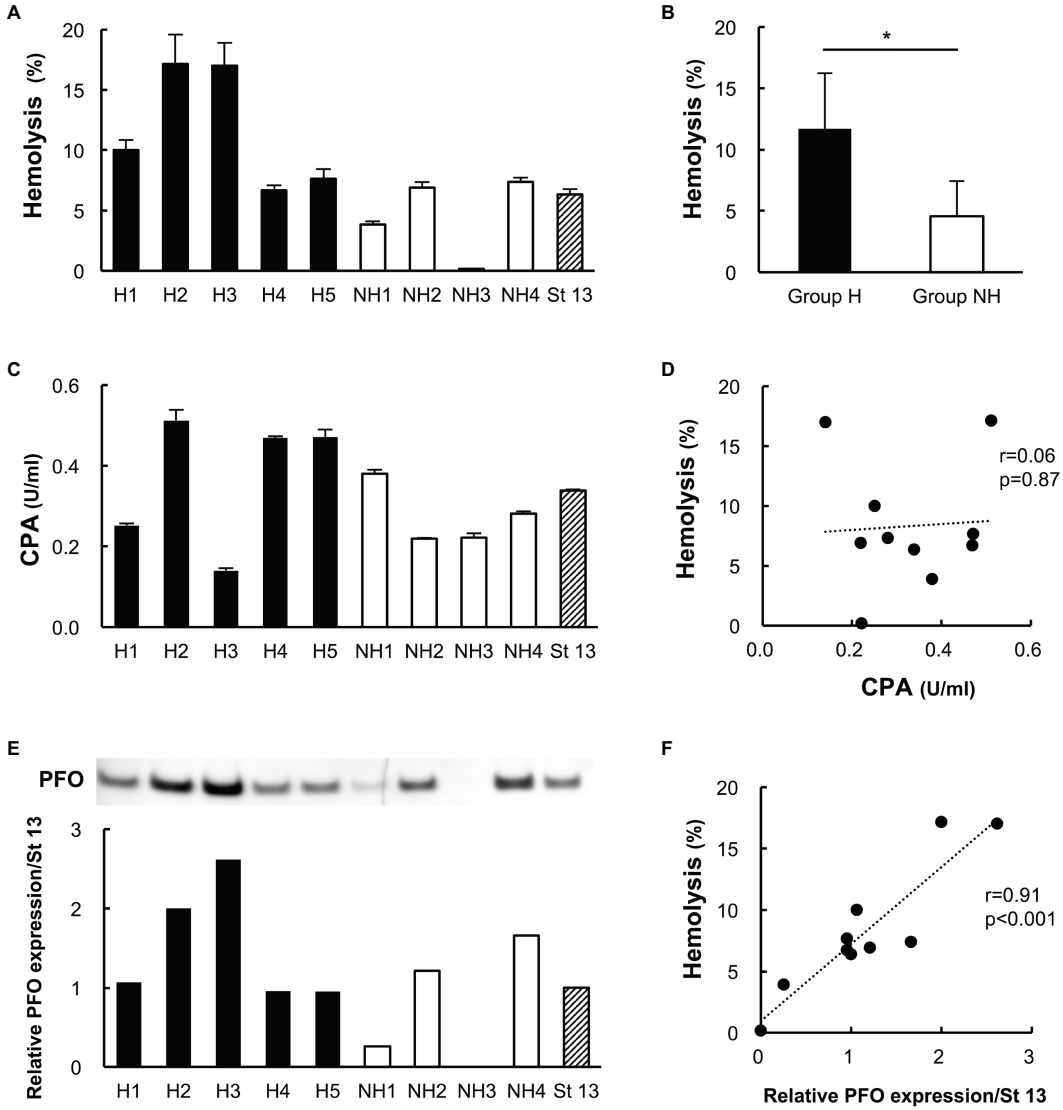
Hemolytic effects on human erythrocytes and the production of phospholipase C (CPA) and perfringolysin O (PFO) in culture supernatants of clinical isolates. Five strains in group H and four strains in group NH, except for the two slow-growing strains (NH5 and NH6), were studied. Strain 13 (St 13), a standard strain, was also examined for comparison. **(A)** Hemolytic effect of culture supernatants of clinical isolates on a 40% human erythrocyte suspension. The hemolysis rate of each strain was calculated according to a formula. The solid bars show strains in group H, the open bars show strains in group NH, and the diagonal striped bar shows strain 13. The values are the average of three independent experiments conducted with technical triplicates, and the error bars indicate the SDs. **(B)** Hemolytic effect of group H (*n* = 5) and group NH (*n* = 4) strains on a 40% human erythrocyte suspension. ^*^p < 0.05 **(C)** CPA concentration in culture supernatants of clinical isolates. The values are the average of three independent experiments conducted with technical duplicates, and the error bars indicate the SDs. **(D)** Correlation between the hemolysis rate and CPA production. The hemolysis rate is plotted against the CPA concentration. **(E)** Expression of PFO in culture supernatants of clinical isolates, as determined by Western blot analysis. Relative PFO expression normalized to that of strain 13 is expressed numerically. **(F)** Correlation between the hemolysis rate and PFO production. The hemolysis rate is plotted against the relative PFO expression level normalized to that of strain 13.

Compared to those of group NH strains, the culture supernatants of group H strains clearly hemolyzed the 40% washed human erythrocyte solution (group H 12.2 ± 5.4% vs. group NH 4.4 ± 3.2%, *p* < 0.05; Figures 2A,B).

### Production of CPA in Culture Supernatants of Clinical Isolates

Phospholipase C was detected in the supernatants of all clinical isolates in both groups. The concentration ranged from 0.14 to 0.51 U/ml, and CPA production did not differ significantly between the two groups (group H strains 0.37 ± 0.16 U/ml vs. group NH strains 0.28 ± 0.08 U/ml, *p* = 0.33; Figure 2C). The hemolysis rate was not correlated with PCA production by the isolated strains (*r* = 0.06; *p* = 0.87, Figure 2D).

### Expression of PFO in Culture Supernatants of Clinical Isolates

Perfringolysin O expression tended to be higher in group H strains than in group NH strains, although the difference was nonsignificant (group H strains 1.52 ± 0.68 vs. group NH strains 0.78 ± 0.68, *p* = 0.19; Figure 2E). PFO expression in preparations of strains H2 and H3, which had high hemolysis rates against 40% washed human erythrocytes, was clearly high; in contrast, the NH1 strain, with a low hemolysis rate, had low PFO expression, and the NH3 strain, which did not exhibit hemolysis, did not express PFO (Figure 2E). The higher the PFO expression was, the stronger the hemolytic effect, and the hemolysis rate and PFO expression level were positively correlated (*r* = 0.91, *p* < 0.001; Figure 2F).

### Cytotoxicity of Culture Supernatants of Clinical Isolates in Human PBMCs

**Figure 3:**
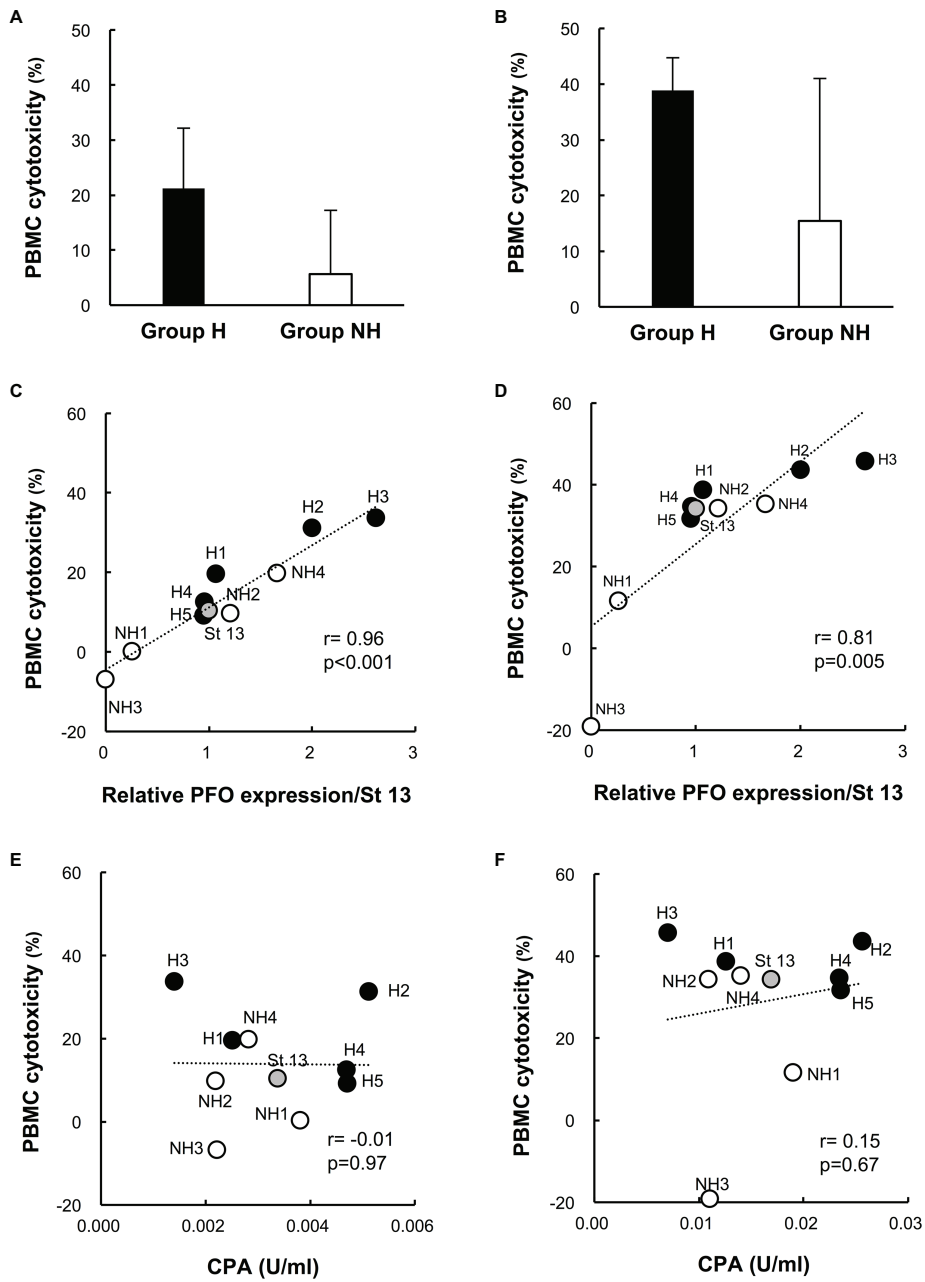
Cytotoxicity of culture supernatants of clinical isolates in human peripheral blood mononuclear cells (PBMCs). **(A)** Cytotoxicity of 1% culture supernatants from group H (*n* = 5) and group NH (*n* = 4) strains in human PBMCs for 4 h. **(B)** Cytotoxicity of 5% culture supernatant in human PBMCs for 4 h. The values are the average of five independent experiments conducted with technical triplicates, and the error bars indicate the SDs. **(C)** Correlation between cytotoxicity in human PBMCs and PFO production in 1% culture supernatants. The average cytotoxicity in PBMCs from five healthy donors is plotted against the relative PFO expression normalized to that of strain 13. **(D)** Correlation between cytotoxicity in human PBMCs and PFO production in 5% culture supernatants. **(E)** Correlation between cytotoxicity in human PBMCs and CPA production in 1% culture supernatants. The average cytotoxicity in PBMCs from five healthy donors is plotted against the CPA concentration. **(F)** Correlation between cytotoxicity in human PBMCs and CPA production in 5% culture supernatants.

The cytotoxicity of group H strain culture supernatants in human PBMCs tended to be higher than that of group NH strains (cytotoxicity of 1% culture supernatant: group H strains 21.2 ± 11.0% vs. group NH strains 5.6 ± 11.6%, *p* = 0.086; cytotoxicity of 5% culture supernatant: group H strains 38.9 ± 5.9% vs. group NH strains 15.4 ± 25.6%, *p* = 0.17; Figures 3A,B). Next, the relationship between the mean PBMC cytotoxicity and relative PFO expression level was examined by treating human PBMCs with the culture supernatant of each clinical isolate at concentrations of 1 and 5%. Cytotoxicity in PBMCs and the PFO expression level were positively correlated for supernatant concentrations of both 1 (Figure 3C) and 5% (Figure 3D; 1%: *r* = 0.96, *p* < 0.001; 5%: *r* = 0.81, *p* = 0.005; Figures 3C,D). In brief, clinical isolates with higher PFO expression exhibited higher cytotoxicity in PBMCs, and group H strains exhibited higher PFO expression and cytotoxicity in PBMCs than group NH strains (Figures 3C,D). No correlation was found between cytotoxicity in PBMCs and the amount of CPA produced (Figures 3E,F).

### Production of Human IL-6 and IL-8 in Culture Supernatants of Clinical Isolates

**Figure 4:**
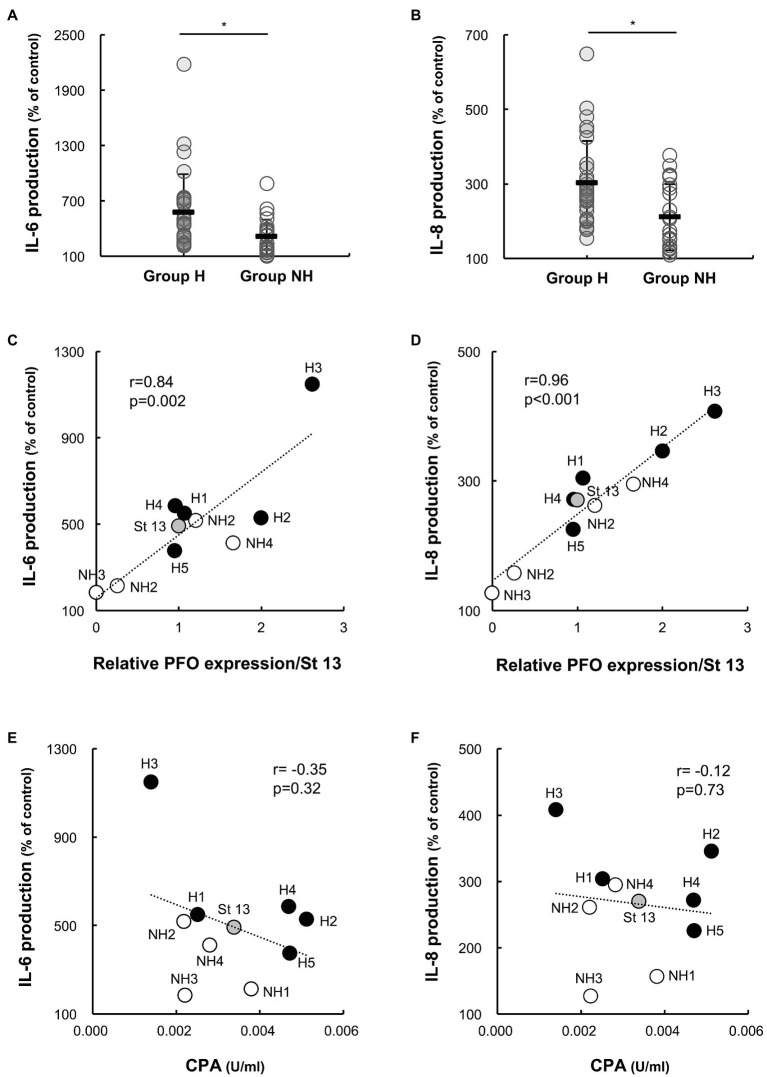
Interleukin-6 (IL-6) and interleukin-8 (IL-8) production by PBMCs induced by supernatants of clinical isolates. Human PBMCs collected from five healthy volunteers on the day of the experiment were stimulated with 1% supernatants of clinical isolates for 4 h. Gifu anaerobic medium (GAM) broth (1%) was used as the control. The graphs show the percentages of IL-6 **(A)** and IL-8 **(B)** production normalized to the PBMC count in group H (*n* = 25) and group NH (*n* = 20) compared to the control. ^*^p < 0.01 **(C)** Correlation between IL-6 production and PFO expression. The average amount of IL-6 produced in five healthy donors is plotted against relative PFO expression normalized to that of strain 13. **(D)** Correlation between IL-8 production and PFO production. The average amount of IL-8 produced in five healthy donors is plotted against relative PFO expression normalized to that of strain 13. **(E)** Correlation between IL-6 production and CPA production. The average amount of IL-6 produced in five healthy donors is plotted against CPA production. **(F)** Correlation between IL-8 production and CPA production. The average amount of IL-8 produced in five healthy donors is plotted against CPA production.

The production of IL-6 and IL-8 in individual preparations was significantly higher for group H strains than for group NH strains (IL-6 production: group H strains 636.9 ± 429.0%, *n* = 25 vs. group NH strains 331.4 ± 196.6%, *n* = 20; *p* < 0.01; IL-8 production: group H strains 310.1 ± 122.0%, *n* = 25 vs. group NH strains 210.2 ± 89.1%, *n* = 20; *p* < 0.01; Figures 4A,B). In addition, the relationships between IL-6 and IL-8 production by PBMCs induced by culture supernatants of *C. perfringens* strains and the relative PFO expression level were examined. Both IL-6 and IL-8 production were significantly correlated with the relative PFO expression level (IL-6: *r* = 0.84, *p* = 0.002; IL-8: *r* = 0.96, *p* < 0.001; Figures 4C,D). Next, the relationships between IL-6 and IL-8 production by PBMCs induced by culture supernatants and the amount of CPA in the culture supernatants were examined. Neither IL-6 production (Figure 4E) nor IL-8 production (Figure 4F) was correlated with the amount of CPA (IL-6: *r* = −0.35, *p* = 0.32; IL-8: *r* = −0.12, *p* = 0.73).

### Effect of rPFO and rCPA on Hemolysis, Cytotoxicity in PBMCs, and Production of IL-6 and IL-8

**Figure 5:**
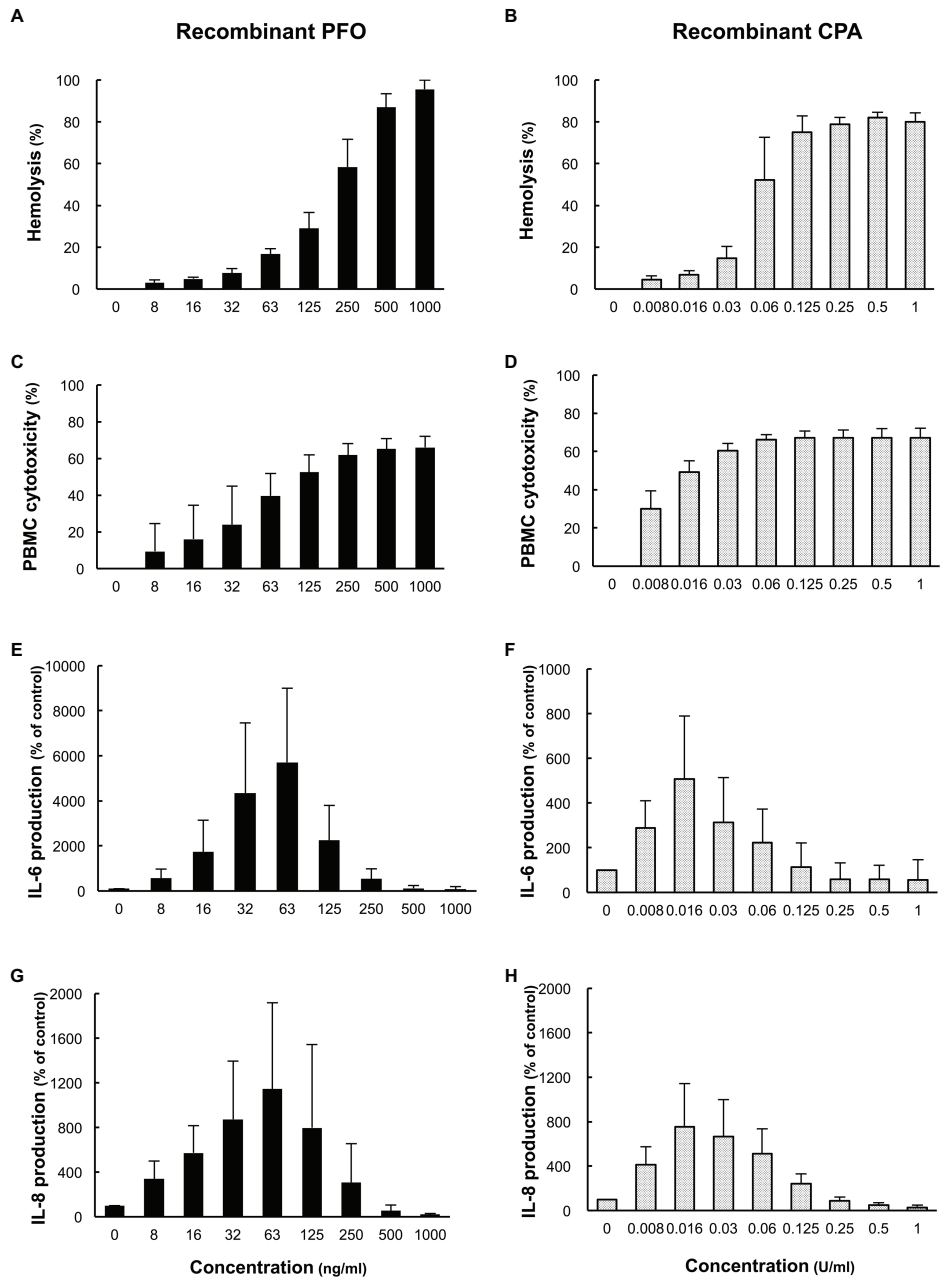
Effects of recombinant PFO (rPFO) and recombinant CPA (rCPA) on human blood cells. The effects of rPFO and rCPA were measured using human blood cells collected from five healthy volunteers on the day of the experiment. **(A)** Hemolytic effect of rPFO on a 40% human erythrocyte suspension. **(B)** Hemolytic effect of rCPA on a 40% human erythrocyte suspension. **(C)** Cytotoxicity of rPFO in human PBMCs. **(D)** Cytotoxicity of rCPA in human PBMCs. **(E)** IL-6 production by human PBMCs induced by rPFO. **(F)** IL-6 production by human PBMCs induced by rCPA. **(G)** IL-8 production by human PBMCs induced by rPFO. **(H)** IL-8 production by human PBMCs induced by rCPA. The values are the average of five independent experiments conducted with technical duplicates, and the error bars indicate the SDs.

The effects of rPFO and rCPA on hemolysis, cytotoxicity in PBMCs, and the production of IL-6 and IL-8 were examined. rPFO increased the human erythrocyte hemolysis rate and cytotoxicity in PBMCs in a concentration-dependent manner (Figures 5A,C) and stimulated IL-6 and IL-8 production most potently at 63 ng/ml (Figures 5E,G). rCPA increased the hemolysis rate and cytotoxicity in PBMCs in a concentration-dependent manner (Figures 5B,D) and stimulated IL-6 and IL-8 production most potently at 16 mU/ml (Figures 5F,H).

### Production of Various Cytokines From Human PBMCs by rPFO and rCPA

**Figure 6:**
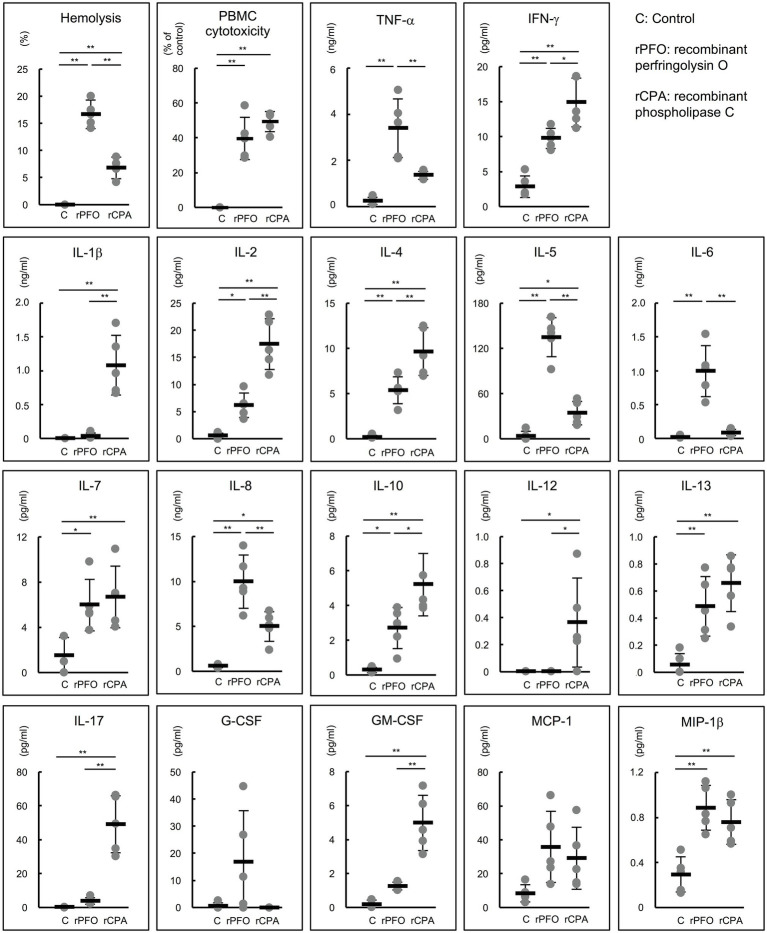
Effects of rPFO and rCPA on human blood cells and the production of various cytokines. The effects of rPFO (63 ng/ml) and rCPA (0.016 U/ml) on human blood cells were evaluated using blood samples from five healthy volunteers. The same volume of phosphate buffered saline (PBS) was used as the control. The black bars indicate the average of five independent experiments conducted with technical duplicates, and the error bars indicate the SDs. ^**^p < 0.01 and ^*^p < 0.05.

The production of various proinflammatory cytokines from human PBMCs stimulated by rPFO and rCPA was comprehensively analyzed using a cytokine assay kit. rPFO and rCPA exhibited almost the same cytotoxicity in PBMCs (rPFO 39.7 ± 12.2 vs. rCPA 49.4 ± 5.8%, *p* = 0.15). rPFO stimulated significantly higher production of tumor necrosis factor-α (TNF-α), interferon-γ (IFN-γ), IL-2, IL-4, IL-5, IL-6, IL-7, IL-8, IL-10, IL-13, and macrophage inflammatory protein-1β (MIP-1β) than the PBS control. rCPA stimulated significantly higher production of IFN-γ, IL-1β, IL-2, IL-4, IL-5, IL-7, IL-8, IL-10, IL-12, IL-13, IL-17, granulocyte macrophage colony-stimulating factor (GM-CSF), and MIP-1β than the control. Specifically, rPFO stimulated the production of TNF-α, IL-5, IL-6, and IL-8 much more potently than rCPA (Figure 6).

## Discussion

Since sepsis with MIH has a fulminant course from early onset, the hemolysis rate of the 40% washed human erythrocyte suspension and production of CPA and PFO by the clinical isolates were compared between the two groups in early logarithmic phase. PFO production was higher in the group H strains (Figure 2E), and the amount of PFO produced was significantly correlated with the hemolysis rate (Figure 2F). CPA also induces hemolysis alone (Kiu and Hall, 2018; Liu et al., 2020); however, CPA production and the hemolysis rate were not correlated. CPA, which hydrolyzes sphingomyelin and lecithin, can lyse erythrocyte, platelet, and endothelial cell membranes (Uzal et al., 2014; Kiu and Hall, 2018; Chinen, 2020). Serum CPA activity was detected in two case reports of *C. perfringens* septicemia with MIH (Moore et al., 1976; Hübl et al., 1993). Therefore, CPA has been considered the most important pathogenic factor for MIH in *C. perfringens* sepsis (Hübl et al., 1993; Chinen, 2020). However, when patient sera with detected CPA activity were used, no hemolytic effect was observed *in vitro* after culture at 37°C for 24 h (Moore et al., 1976). In our study, the amounts of CPA in the culture supernatants of strains H3 and NH3 were 0.14 and 0.22 U/ml, respectively (Figure 2C). Strain H3, which produced PFO, hemolyzed 40% washed human erythrocytes, but strain NH3, which did not produce PFO, did not, suggesting a correlation between PFO expression and hemolysis. These results suggest that PFO is a major hemolytic factor in *C. perfringens* sepsis with MIH.

Next, the effects of *C. perfringens* culture supernatants on human PBMCs were compared between the two groups. The culture supernatants of group H strains, containing more PFO, tended to be more cytotoxic to PBMCs (Figures 3A,B), and PFO expression in the culture supernatant was significantly correlated with cytotoxicity in PBMCs (Figures 3C,D). In addition to causing intravascular hemolysis, PFO was also found to be cytotoxic to PBMCs. The production of IL-6 and IL-8 induced by 1% culture supernatants was clearly higher for the group H strains than the group NH strains (Figures 4A,B), and PFO expression was significantly correlated with the production of both IL-6 and IL-8 (Figures 4C,D). Moreover, rPFO increased the hemolysis rate of 40% washed human erythrocytes, cytotoxicity in PBMCs, and production of IL-6 and IL-8 (Figures 5A,C,E,G). Toxin concentrations in the host differ based on the growth of *C. perfringens*, and toxins are thought to exert their effects at a specific effective concentration (Vidal et al., 2009). Since extensive intravascular hemolysis is detected beginning in the early stage of sepsis with MIH, the effects of toxins in culture supernatants of clinical strains in early logarithmic growth phase were examined herein. PFO in the culture supernatants promoted the hemolysis of 40% washed human erythrocytes, cytotoxicity in PBMCs, and the production of IL-6 and IL-8.

Perfringolysin O is a member of the cholesterol-dependent cytolysin (CDC) family of pore-forming toxins on cholesterol-containing membranes (Popoff, 2014). PFO is thought to be necessary for bacterial escape from macrophage phagosomes in cooperation with CPA (O’Brien and Melville, 2004). PFO was reported to augment the action of CPA and to act synergistically with CPA to contribute to the enhanced pathogenesis of gas gangrene in humans (Bryant et al., 1993; Awad et al., 2001; Uzal et al., 2014; Verherstraeten et al., 2015). PFO was also reported to cause synergistic effects with other toxins and contribute to the progression of animal diseases, including bovine necrohemorrhagic enteritis (in cooperation with CPA; Verherstraeten et al., 2013) and enterotoxemia of sheep and goats (in cooperation with ε-toxin; Verherstraeten et al., 2015). Despite the widespread presence of PFO-producing *C. perfringens* strains, no disease has been identified in which PFO is a major virulence factor (Bryant et al., 1993; Verherstraeten et al., 2015; Kiu and Hall, 2018). This study provided the first demonstration that PFO is one of the major pathogenic toxin promoting the development of sepsis with MIH after *C. perfringens* infection. Further experiment using modified strains deficient in the production of PFO or CPA will be needed to finally prove that PFO is the major pathogenic factor for MIH in *C. perfringens* septicemia.

Furthermore, the effect of PFO on the induction of proinflammatory cytokine production by human PBMCs was investigated. rPFO significantly increased the production of TNF-α, IFN-γ, IL-2, IL-4, IL-5, IL-6, IL-7, IL-8, IL-10, IL-13, and MIP-1β, and its effects on TNF-α, IL-5, IL-6, and IL-8 were particularly strong compared with those of rCPA. rCPA also significantly enhanced the production of various proinflammatory cytokines (Figure 6). Both toxins had very strong activity to induce the production of proinflammatory cytokines by human PBMCs. PFO is speculated to induce intravascular hemolysis and the production of proinflammatory cytokines, such as TNF-α, IL-5, IL-6, and IL-8 beginning in the early stage of infection in hosts infected with *C. perfringens*. As SIRS is caused by overproduction of IL-6 (Kang et al., 2014), *C. perfringens* strains with high PFO production and strong IL-6 inducibility might lead more readily to SIRS than those producing only CPA. In other words, the effects of PFO on human erythrocytes and PBMCs could explain why the pathogenesis of sepsis with MIH follows a rapidly progressive, lethal course. In addition, TNF-α and IL-6, which are strongly induced by PFO, are involved in the development of pathological pain as well as fever (Zhang and An, 2007), and IL-6, IL-8, and IL-10 are reported to cause acute lung injury/ARDS (Aisiku et al., 2016; Yang et al., 2020). Many patients with sepsis with MIH have severe pain and acute lung injury/ARDS from early onset, suggesting that the action of both toxins initiates the clinical pathogenesis of fulminant sepsis with MIH.

In this study, PFO-induced cytokine production was measured using whole PBMCs. One report indicated that 50 ng/ml PFO induced the secretion of TNF-α and IL-6 from mouse bone marrow-derived macrophages *via* Toll-like receptor 4 (Park et al., 2004). The specific cells within human PBMCs that are involved in the production of each cytokine induced by PFO must be clarified. Patients with *C. perfringens* sepsis with MIH are seriously ill, and systemic administration of potent antimicrobial agents and resection of the lesion are needed early in the disease course (Simon et al., 2014). Many patients are already in shock by the time this disease is suspected, and many die prior to any therapeutic effect (van Bunderen et al., 2010; Chinen, 2020). In general, antimicrobial therapy alone is often inadequate in patients with *Clostridium* infections, and antitoxin therapy has already been established for patients with other *Clostridium* infections (*C. tetani* and *C. botulinum* infections; Johnson, 1997). In newborn piglets with *C. perfringens* type C infection, an anti-β toxoid vaccine has been reported to be effective against necrotizing enterocolitis (Richard et al., 2019). The development of a PFO antitoxin and cytokine-targeted therapies using an anti-IL-6 antibody (Kang et al., 2014) should be considered for the treatment of patients with *C. perfringens* sepsis with MIH.

## Data Availability Statement

The original contributions presented in the study are included in the article/Supplementary Material, further inquiries can be directed to the corresponding author.

## Ethics Statement

The study using clinical isolates was approved by the Ethical Committee of Nihon University Hospital (Number 20160401). The studies involving human participants were reviewed and approved by the Institutional Review Board of Nihon University School of Medicine (Number 30-13-1). The patients/participants provided their written informed consent to participate in this study.

## Author Contributions

AS conceived the study with input from KO, SK-A, SK, and SH, contributed to the procurement of clinical isolates and analysis of the clinical profiles, and wrote the original draft of the manuscript. AS, KO, SK-A, and AM contributed to data collection, data analysis, and data interpretation. KO, SK-A, and SK reviewed and edited the manuscript. SH supervised this study. All authors contributed to the article and approved the submitted version.

## Conflict of Interest

AM and SK are employed by Miyarisan Pharmaceutical Company.

The remaining authors declare that the research was conducted in the absence of any commercial or financial relationships that could be construed as a potential conflict of interest.

## Publisher’s Note

All claims expressed in this article are solely those of the authors and do not necessarily represent those of their affiliated organizations, or those of the publisher, the editors and the reviewers. Any product that may be evaluated in this article, or claim that may be made by its manufacturer, is not guaranteed or endorsed by the publisher.
