# Supplementary material for: Pathogenic Characterization of Clostridium perfringens Strains Isolated From Patients With Massive Intravascular Hemolysis
Source: Front Microbiol. 2021 Jul 27;12:713509. doi: 10.3389/fmicb.2021.713509 (PMC8353389; doi:10.3389/fmicb.2021.713509)
Supplement: Supplementary file 1 [file Data_Sheet_1.PDF]

Table S1 Primers used in this study

Primer name	Gene name	Primer sequence
CPE0030-1	hlyA	TGGTGATGGTGGAGTTGCCT
CPE0030-2		CTCTCATGTATTCTGTTACA
CPE0036-1	plc	TTTCCTGGGTTGTCCATTTC
CPE0036-2		AGTCTACGCTTGGGATGGAA
CPE0163-1	pfoA	TGTAGCTTATGGAAGAACTA
CPE0163-2		CACCATTCCCAAGCAAGACC
CPE0173-1	colA	CCTGATGAATTTTTTCCACCAAA
CPE0173-2		GGATATGATGCTAAAAACACTGAGTTCTAT
CPE0191-R	nagH	GTCTCATTCCCCACCTGAGC
CPE0191-F		TAAAAAGCGGGTTGATGAAG
CPE0437-1	hlyB	TTGCAGCTGCAGAGATGGCA
CPE0437-2		GACTTCTCCCCTAGGAGTCA
CPE0452-1	entC	GAGTTAAACGGACAAAAAGG
CPE0452-2		TCTTTAGAAATCATCTCAGT
CPE0553-1	nanJ	TACAGAGGGCGGAAGTGGAT
CPE0553-2		ACTACTTGGCACTCTGTC
CPE0606-1	entD	TGGAAGGGGTGGCATGTTGG
CPE0606-2		GCTTCCTGGGCCGCTTCTCA
CPE0725-R	nanI	CCCTCCATCTTCACTTCT
CPE0725-F		TACTTTAGACTCAGGGAAAA
CPE0737-1	fbpA	GTTCCTTGCTCTGGATTAGA
CPE0737-2		GGTACCCTAAGAAACTGTAC
CPE0845-R	virT	GGT ACT TTC TTT ACG TTA CC
CPE0845-F		CTA CAC ATA CCT GAA TCT TG
CPE0846-1	ccp	CCA ATG TGG TAT TGC TTG TC
CPE0846-2		TGG GAA AAG TGA ATT TCC AG
CPE0881-R	nagI	GCTTCTCCCTCTTCTCCCTC
CPE0881-F		GAATCAGAAATTGAGGGGGC
CPE0957-1	vrr	TGA AAC ATA CAA AAA GGA TT
CPE0957-2		TAC AAT TAT GGA ATA TGC AA
CPE1234-R	nagJ	CTGGATATGGCTCTCTCCAT
CPE1234-F		GGTGAGGAAGAGGCAGATGA
CPE1258-1	entA	GCAGGTGAATATCACACAAT
CPE1258-2		CGCCAAGAGGAGTAACAGAA
CPE1264-1	sialidase-like	ACCCCTTCCTTAGTAGCCTC
CPE1264-2		AGGAGAATTCACAGATGGAG
CPE1279-R	nagK	GGCACTGTAACTTTAGGGAC
CPE1279-F		GGCTCTCTCCACTTAAGACT
CPE1354-1	entB	TGATCCCAAGTTGTTCTTCC
CPE1354-2		ACTCAGGAATGGATACTTCA
CPE1368-1	cadA	CCTGAATCATTTCCTGATCC
CPE1368-2		CTCAGTCAGAGGCTGTAGAA
CPE1474-1	hlyC	GGCACTAACAGCTAAAGCTA
CPE1474-2		ACTCCACCTATTGCTAACCA
CPE1523-R	nagL	TCTGCTCCCGCCTTAGCAAG
CPE1523-F		AGA GGG AGA GGT TAA CAT AG
CPE1818-1	hlyD	AGGAGAGAAATCAAGACCTA
CPE1818-2		AAAAGGTTCCTGTAAGCTCA
CPE1847-1	fbpB	AACAGGTGGAAGAATAATTG
CPE1847-2		TCTTTGAATATCTGCACTTT
CPE1882-1	collagenase-like	TGTATATGTGTGATCTACAG
CPE1882-2		AAGACTGGTGATATGGTTCA
CPE1915-1	hlyE	TGCCAGATAGCATGATTGTA
CPE1915-2		ACCCACGGAATTGGAGTGGT
PCP17-1	cpb2	AGGAAATCGACGCTTATAGA
PCP17-2		TGTCTAGCAGAATCAGGGTT
PCP57-1	cna	TGC AGA AGC AAC TTC ACA AG
PCP57-2		TTG TAT AGG AGA GTT CTT AA
CPE-1	cpe	AGA TGG TTG GAT ATT AGG GG
CPE-2		TTA CTC CAT CAC CTA AGG ACT
delta-1	delta-toxin	TACCAAATGATTTAGGGAGT
delta-2		ATCCGTCACGCGTTTCGGCA

Table S2 Gene variation of possible virulence factors

Gene name	CPE0030 *hlyA*	CPE0036 *plc*	CPE0163 *pfoA*	CPE0173 *colA*	CPE0191 *nagH*	CPE0437 *hlyB*	CPE0452 *entC*	CPE0553 *nanJ*	CPE0606 *entD*	CPE0725 *nanI*	CPE0737 *fbpA*	CPE0845 *virT*	CPE0846 *ccp*	CPE0881 *nagI*	CPE0957 *vrr*
**H1**	+	+	+	+	+	+	-	+	-	+	+	+	+	+	+
**H2**	+	+	+	+	+	+	-	+	+	+	+	+	+	+	+
**H3**	+	+	-	+	+	+	-	+	+	-	+	+	+	+	+
**H4**	+	+	+	+	+	+	-	+	+	+	+	+	+	+	+
**H5**	+	+	+	+	+	+	-	+	+	+	+	-	+	+	+
**NH1**	+	+	+	+	+	+	-	+	+	+	+	+	+	+	+
**NH2**	+	+	+	+	+	+	-	+	+	+	+	-	+	+	+
**NH3**	+	+	+	+	+	+	-	+	+	+	+	+	+	+	+
**NH4**	+	+	+	+	+	+	-	+	+	+	+	+	+	+	+
**NH5**	+	+	+	+	+	+	-	+	+	+	+	-	+	+	+
**NH6**	+	+	+	+	+	+	-	+	+	+	+	+	+	+	+
**strain 13**	+	+	+	+	+	+	+	+	+	+	+	+	+	+	+

Gene name	CPE1234 *nagJ*	CPE1258 *entA*	CPE1264 *sial-like*	CPE1279 *nagK*	CPE1354 *entB*	CPE1368 *cadA*	CPE1474 *hlyC*	CPE1523 *nagL*	CPE1818 *hlyD*	CPE1847 *fbpB*	CPE1882 *collagenase*	CPE1915 *hlyE*	PCP17 *cpb2*	PCP57 *cna*	CPE *cpe*	delta *delta-toxin gene*
**H1**	+	+	+	+	+	-	+	+	+	+	+	+	-	+	-	+
**H2**	+	+	+	+	+	-	+	+	+	+	+	+	-	+	-	-
**H3**	+	+	+	+	+	-	+	-	+	-	+	+	+	-	-	-
**H4**	+	+	+	+	+	+	+	+	+	+	+	+	+	+	+	-
**H5**	+	+	+	+	+	-	+	-	+	+	+	+	-	+	-	-
**NH1**	+	+	+	+	+	+	+	+	+	+	+	+	-	+	+	-
**NH2**	+	+	-	+	+	-	+	-	+	+	+	+	-	+	-	+
**NH3**	+	+	+	+	+	+	+	+	+	+	+	+	-	+	+	-
**NH4**	+	+	+	-	+	+	+	+	+	+	+	+	-	+	-	-
**NH5**	+	+	+	+	+	-	+	-	+	+	+	+	-	+	-	-
**NH6**	+	+	+	+	+	-	+	-	+	+	+	+	-	+	-	-
**strain 13**	+	+	+	+	+	+	+	+	+	+	+	+	+	+	-	-
